# Requirement for Drosophila SNMP1 for Rapid Activation and Termination of Pheromone-Induced Activity

**DOI:** 10.1371/journal.pgen.1004600

**Published:** 2014-09-25

**Authors:** Zhengzheng Li, Jinfei D. Ni, Jia Huang, Craig Montell

**Affiliations:** 1 Department of Biological Chemistry, The Johns Hopkins University School of Medicine, Baltimore, Maryland, United States of America; 2 Neuroscience Research Institute and Department of Molecular, Cellular and Developmental Biology, University of California, Santa Barbara, Santa Barbara, California; The University of North Carolina at Chapel Hill, United States of America

## Abstract

Pheromones are used for conspecific communication by many animals. In Drosophila, the volatile male-specific pheromone 11-*cis* vaccenyl acetate (cVA) supplies an important signal for gender recognition. Sensing of cVA by the olfactory system depends on multiple components, including an olfactory receptor (OR67d), the co-receptor ORCO, and an odorant binding protein (LUSH). In addition, a CD36 related protein, sensory neuron membrane protein 1 (SNMP1) is also involved in cVA detection. Loss of SNMP1 has been reported to eliminate cVA responsiveness, and to greatly increase spontaneous activity of OR67d-expressing olfactory receptor neurons (ORNs). Here, we found the *snmp1^1^* mutation did not abolish cVA responsiveness or cause high spontaneous activity. The cVA responses in *snmp1* mutants displayed a delayed onset, and took longer to reach peak activity than wild-type. Most strikingly, loss of SNMP1 caused a dramatic delay in signal termination. The profound impairment in signal inactivation accounted for the previously reported “spontaneous activity,” which represented continuous activation following transient exposure to environmental cVA. We introduced the silk moth receptor (BmOR1) in OR67d ORNs of *snmp1^1^* flies and found that the ORNs showed slow activation and deactivation kinetics in response to the BmOR1 ligand (bombykol). We expressed the bombykol receptor complex in Xenopus oocytes in the presence or absence of the silk moth SNMP1 (BmSNMP) and found that addition of BmSNMP accelerated receptor activation and deactivation. Our results thus clarify SNMP1 as an important player required for the rapid kinetics of the pheromone response in insects.

## Author Summary

Pheromones are chemicals produced and released by animals for social communication with other members of their species. For example, male fruit flies produce a volatile pheromone that is sensed by both males and females, and which functions in gender recognition. This volatile male pheromone, called 11-*cis* vaccenyl acetate, is detected by olfactory neurons housed in hair-like appendages on the insect antenna. To effectively sense the pheromone, especially during navigation, the olfactory neurons must respond rapidly, and then quickly inactivate after the stimulation ceases. We found that a CD36-related protein referred to as sensory neuron membrane protein 1 (SNMP1) was required by olfactory neurons for the rapid on and off responses to 11-*cis* vaccenyl acetate. Loss of SNMP1 reduced the initial sensitivity to the pheromone, and then caused a strikingly slower termination of the response after removal of the pheromone. Our findings demonstrate that SNMP1 is a critical player that allows olfactory neurons to achieve sensitive and rapid on and off responses to a pheromone that is critical for social interactions in insects.

## Introduction

Pheromones are chemicals that trigger or inhibit stereotyped social behaviors, such as aggregation, courtship and mating [1], [2], [3]. Studies on insects have contributed enormously to our understanding of pheromones [2], [3]. The first pheromone characterized was bombykol— a volatile 16-carbon alcohol synthesized in the female gland of the silk moth, *Bombyx mori* [4], [5]. Male silk moths use bombykol as a navigation cue to find female mates, and this pheromone can be sensed over long distances [5], [6]. Volatile pheromones are typically comprised of hydrocarbon chains [7], and are perceived by olfactory receptor neurons (ORNs) in the antenna of insects. One such pheromone, 11-*cis* vaccenyl acetate (cVA), represents the only volatile pheromone known in the fruit fly, *Drosophila melanogaster*. This chemical is released from the ejaculatory bulb of the males [8] and is sensed by both males and females, the latter of which receive the pheromone during copulation [9]. The ORNs that sense the volatile cVA signal are housed in one type of olfactory hair on the antenna (trichoid sensilla), referred to as T1 sensilla [10]. Detection of cVA modifies a host of behaviors including male-male aggression, social aggregation, male-female and male-male courtship behavior [11], [12], [13], [14], [15].

Due to the critical roles of pheromone-induced behaviors, the mechanisms underlying insect pheromone detection have been studied extensively. The receptors for cVA (OR67d) and bombykol (BmOR1) belong to the insect olfactory receptor (OR) family [16], [17], [18]. Another OR, referred to as ORCO, is conserved in many insects, and in Drosophila serves as a co-receptor, which is broadly required for trafficking and function of other ORs [19], [20], [21].

Because pheromones are hydrophobic, their solubility depends in part on association with odorant-binding proteins (OBPs) or pheromone-binding proteins (PBPs) present in the endolymph of the sensilla [22], [23]. In Drosophila, LUSH is the OBP required for sensation of cVA [24]. Upon binding cVA, LUSH has been reported to undergo a conformational change, which in turn activates OR67d [25]. However, another study concludes that cVA directly activates the receptor [26].

SNMP1, which is a member of the CD36-scavenger family, also contributes to the pheromone response [27], [28]. Mutations disrupting this integral membrane protein have been reported to eliminate cVA detection [27], [28]. SNMP1 is expressed in the antenna in the dendrites of trichoid ORNs [27], [28], consistent with its role in cVA detection. Loss of SNMP1 also causes a dramatic increase in spontaneous activity of T1 ORNs [27], [28]; although, the mechanism underlying the increased spontaneous activity is unknown.

Here, we found that loss of SNMP1 did not eliminate cVA-evoked activity, and was required for fast inactivation. The onset of the cVA-induced action potentials was delayed, and the activity increased slowly. Following cessation of the cVA stimulus, the activity continued for many minutes. This contrasted with the wild-type response, which terminated in less than a second. Thus, inactivation was delayed dramatically. We also demonstrated that *snmp1^1^* mutant ORNs did not exhibit an increase in spontaneous activity. Rather, the high frequency of action potentials was due to the highly persistent activity initiated by cVA in the environment. We expressed the bombykol receptor from the silk moth (BmOR1 and BmORCO) in Xenopus oocytes, and found that addition of the silk moth SNMP1 significantly increased the kinetics of the activation and inactivation of the receptor. Thus, we conclude that SNMP1 functions in promoting the rapid activation and inactivation of pheromone receptors to achieve fast onset and termination of pheromone sensitive ORNs.

## Results

**Figure 1:**
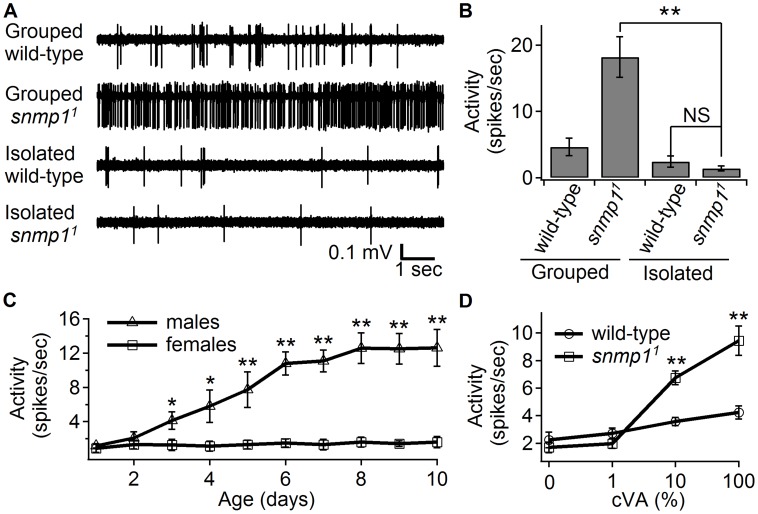
Effects of prior exposure either to males or to cVA on “spontaneous” spiking activity. Single sensillum recordings (SSRs) were from trichoid sensilla (T1), which contain an OR67d-expressing ORN. Neither the wild-type nor the *snmp1^1^* flies were exposed to cVA during the recordings. (A) Representative traces showing firing activities from wild-type and *snmp1^1^* females, which were either reared in isolation or in groups with males, as indicated. (B) Mean firing rates elicited by grouped or isolated wild-type and *snmp1^1^* females. n = 15–18. (C) Average spiking activities of OR67d neurons from isolated male or female *snmp1^1^* flies. The ages of the flies are indicated. n = 8–10. (D) Wild-type and *snmp1^1^* females were exposed to cVA or the vehicle (paraffin oil) for 24 hrs immediately prior to the recordings. n = 16–18. Mean ±S.E.M. The asterisks indicate significant differences between groups (*p<0.05, **p<0.01) using ANOVA with Bonferroni-Holm *post hoc* test to compare multiple samples (B) and the unpaired Student *t*-test for comparing pairs of data (C and D). NS, no significant difference.

To characterize the role of SNMP1 in the cVA response, we performed single sensillum recordings, initially using conditions similar to those described previously [27], [28]. We recorded action potentials from trichoid sensilla (T1), which contain OR67d-expressing ORNs. Consistent with earlier studies [27], [28], the ORNs from *snmp1^1^* females showed high “spontaneous activity” relative to wild-type females (Figure 1A and 1B). The females used in these experiments, and in the previous reports on *snmp^1^*, were raised in groups, which included males and other female flies.

Surprisingly, when we modified the rearing paradigm, and maintained the *snmp1^1^* females in isolation from the pupal stage through adulthood, the high “spontaneous activity” was eliminated, and the frequency of action potentials in the absence of cVA was similar or marginally lower (though not significantly) than in wild-type females (Figure 1A and 1B). We also recorded background action potentials from singly housed *snmp1^1^* mutant males. Young males (≤2 days old) displayed low background activity, similar to females (Figure 1C). In contrast, older mutant males exhibiting higher background activity (Figure 1C). This age-dependent increase did not occur with *snmp1^1^* females (Figure 1C). Because males but not females produce cVA, these findings suggest that cVA released from males induce the background action potentials.

To test whether exposure to environmental cVA caused the high basal activity, we reared *snmp1^1^* females under isolation, and then exposed them to cVA for 24 hours. We then measured action potentials elicited by OR67d ORNs in the absence of any cVA during the electrophysiological measurements. Pre-incubation with 10% or 100% cVA caused the *snmp1^1^* females to show significantly higher activity than the similarly treated wild-type females (Figure 1D). Pretreatment of *snmp1^1^* flies either with the vehicle (paraffin oil; 0% cVA) or with 1% cVA had no significant effect (Figure 1D). These results support the proposal that the elevated activity elicited by the grouped *snmp1^1^* mutants was caused by the environmental cVA derived from male flies.

In addition to T1, the antenna contains other trichoid sensilla that respond to fly odors [29]. To address whether *SNMP1* function was required generally in ORNs for attenuating the activity of environmental fly odors, we recorded the basal activity of T3 sensilla from singly and group housed male and female flies. The action potentials from the T3 sensilla exhibited three size amplitudes (A, B and C), each of which was derived from distinct ORNs (Figure S1A). The frequencies of action potentials from the three different ORNs were indistinguishable between wild-type and *snmp1^1^* males and females, regardless of whether they were individually or group housed (Figure S1B). Thus, all pheromone responsive ORNs in the *SNMP1* mutants do not show higher basal activity in response to pheromone pre-exposure.

**Figure 2:**
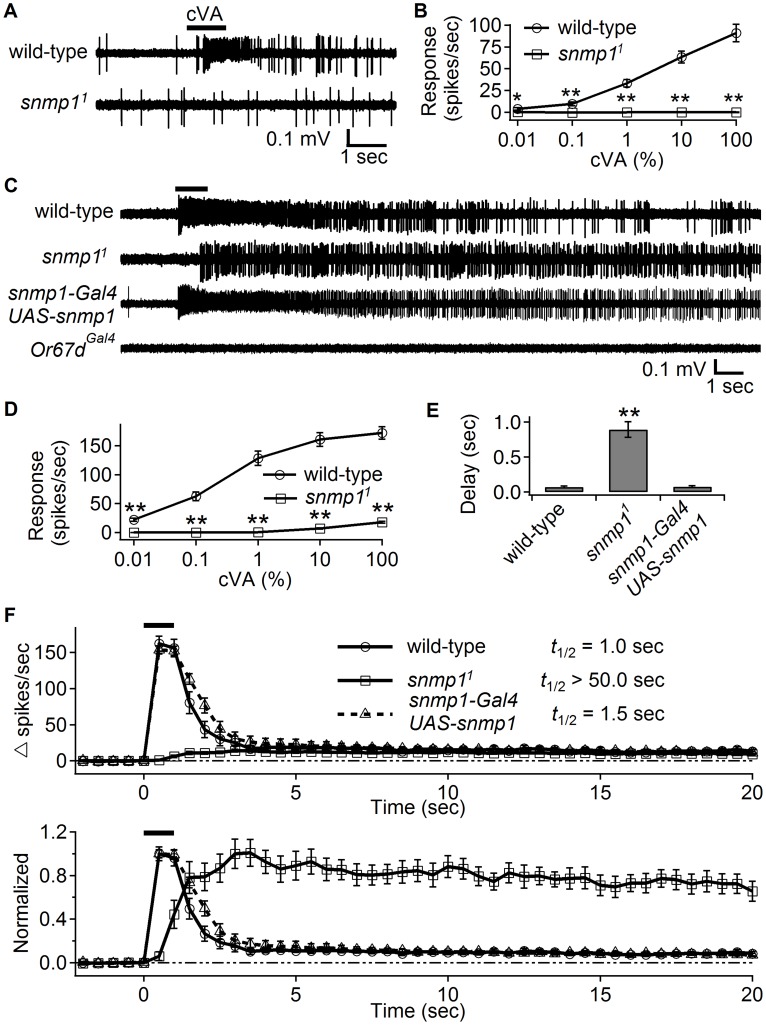
High cVA levels elicited weak responses in *snmp1^1^*, which displayed slow activation and deactivation kinetics. The SSRs were from female flies of the indicated genotypes. (A) Representative SSRs obtained from OR67d ORNs stimulated with cVA for 1 sec (indicated by the horizontal black bar) using the conventional odorant delivery approach. (B) Action potential frequencies as a function of the concentration of the applied cVA using the conventional odorant delivery method. (C) Representative traces showing the responses from OR67d ORNs evoked by close-range application of 100% cVA (indicated by the horizontal bar above the trace). The cVA was puffed onto the antenna through a pipette tip placed 3 mm away from the fly antenna. The flies expressing *UAS*-*snmp1* under the control of the *snmp1-Gal4* transgene were in a *snmp1^1^* background. (D) Quantification of peak firing rates following close-range application of cVA. n = 17–20. (E) Quantification of the onset delays of the responses to close-range stimulation with cVA. (F) The upper graph shows the duration of the firing of OR67d neurons after close-range application of 100% cVA. The estimated times required for a 50% reduction of the evoked firing rates (*t*_1/2_) are shown (n = 17–20). The frequencies (spikes/sec) were binned every 0.5 sec. Therefore, the *t*_1/2_ were rounded to the nearest 0.5 sec. The traces in the lower graph plot were derived from the upper panel, and were normalized to their respective peak firing rates. Means ±S.E.M. The asterisks indicate significant differences from wild-type and rescue flies (**p<0.01) based on unpaired Student *t*-test for comparing pairs of data (B and D) and ANOVA with the Bonferroni-Holm *post hoc* test for comparing multiple samples (E).

A major problem with the hypothesis that the higher background activity in *snmp1^1^* mutants is due to environmental cVA, is that the *snmp1^1^* animals are reported to be completely insensitive to cVA [27], [28]. One possibility was that the insensitivity to cVA was caused by the perpetual high background activity, which caused the OR67d ORNs to be unresponsive to further stimulation. To test this possibility, we stimulated the singly housed *snmp1^1^* females with cVA. However, these animals with low background activity still failed to respond to cVA, even at the highest concentration tested (Figure 2A and 2B).

The preceding results still do not resolve the question as to how environmental cVA could lead to elevated background activity, given that the mutant OR67d ORNs are unresponsive to cVA stimulation during single sensillum recordings. One explanation is that the cVA stimulation is inadequate, and that the *snmp1^1^* flies must be exposed to higher levels of cVA, such as those that might be achieved through close interactions with males [29]. In support of the concept that SNMP1 might not be absolutely required for activation by cVA, ectopic expression of OR67d in ab3A neurons, which lack SNMP1, is sufficient to elicit a response to cVA if it is applied in close proximity to the sensilla [29]. Therefore, instead of using the conventional delivery method, in which cVA was diluted into air that was streamed through a tube, we puffed cVA from a pipette placed in very close proximity to the antenna (close-range application). 100% cVA (1 second) delivered by this close-range application evoked a robust response in wild-type flies (Figure 2C). Of significance here, the *snmp1^1^* females also responded to the cVA application, although not as strongly as wild-type (Figure 2C and 2D). The *snmp1^1^* females elicited responses to 10% and 100% cVA, but not to 1% or lower levels of cVA (Figure 2D). In addition, there was a significant delay in production of the action potentials (Figure 2C and 2E). We rescued these phenotypes by expressing a wild-type *snmp1* transgene (*UAS-snmp1*) under control of the *snmp1-Gal4* (Figure 2C, 2E and 2F). To provide a negative control, we tested *Or67d^Gal4^* mutant females and found that close-range application did not evoke action potentials in these animals (Figure 2C).

**Figure 3:**
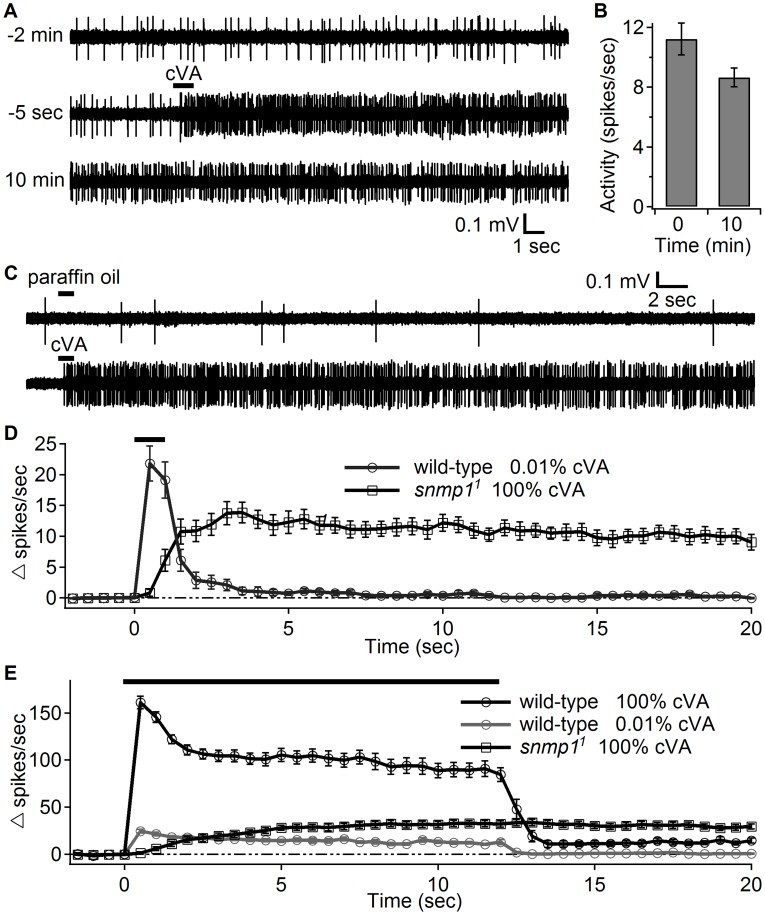
OR67d ORNs in *snmp1^1^* females showed high neuronal activity long after transient stimulation with cVA. (A) Close-range application of cVA elicited long-lasting responses in OR67d ORNs from *snmp1^1^* flies. The upper, middle and lower traces show the traces recorded at different times relative to the cessation of the cVA stimulation The upper trace indicates the spontaneous activity of the OR67d ORNs before cVA stimulation. The middle trace shows the point of application of 100% cVA applied for 1 sec (indicated by the black bar). The start of the cVA puff is defined as time 0. The lower trace demonstrates that the response persisted 10 min after the cVA stimulation. (B) Quantification of the firing rates immediately after cessation of the cVA stimulation (0 min) and 10 min later (n = 5). (C) Close range application of the cVA solvent (paraffin oil) did not elicit responses in OR67d ORNs. (D) Duration of the responses of OR67d ORNs to close-range application of 0.01% (wild-type) and 100% cVA (*snmp1^1^*, regenerated from Fig. 2F). (E) The dynamics of the firing rates after close-range application of with either 100% or 0.01% cVA for 12 sec as indicated by the black bar. Means ±S.E.M.

An additional and pronounced aspect of the *snmp1^1^* phenotype occurred after termination of the cVA stimulus. When we exposed wild-type flies to a transient cVA puff, the spiking activity of wild-type quickly decreased, as the firing declined by 50% in ∼1 second (Figure 2F; *t*_1/2_; the data were binned every 0.5 seconds, resulting in calculations of the *t*_1/2_ to the nearest 0.5 second). In stark contrast, the weaker activity in *snmp1^1^* flies was very long-lasting and showed almost no decline 20 seconds after the cVA puff (Figure 2F; *t*_1/2_>50 seconds). Strikingly, the spiking activity was still robust after 10 minutes (Figure 3A and 3B). Application of the vehicle (paraffin oil) to the *snmp1^1^* fly had no effect (Figure 3C).

It was possible that the deactivation defect exhibited by *snmp1^1^* flies was a manifestation of the weak cVA response. In other words, rapid deactivation might depend on a robust response to the initial stimulus. To address this possibility, we stimulated wild-type flies with a low level of cVA (0.01%) that evoked an initial firing rate comparable to that induced by exposing *snmp1^1^* to a 10,000-fold higher concentration of cVA (100%). Although the evoked firing rates were similar in these wild-type and mutant flies, only the *snmp1^1^* flies exhibited persistent action potentials following removal of cVA (Figure 3D).

We further investigated the slow activation of *snmp1^1^ Or67d* ORNs by presenting a prolonged cVA stimulation (12 seconds). Unlike the response by wild-type flies, which reached the maximum activation within 0.5 seconds during the 100% cVA application, the *snmp1^1^* flies showed a gradual increase in firing activity during the stimulation (Figure 3E; *t_95_* = 7.5 seconds, time to reach 95% of the maximum response; data were binned every 0.5 seconds). Again, this was not a side effect of the weak response, as wild-type flies that displayed a similarly weak response (evoked by 0.01% cVA) also reached the maximum firing rate in the first 0.5 second window after stimulation (Figure 3E). Elongating the stimulation to 20 seconds did not further increase the activity elicited by the 100% cVA stimulation (Figure S2). While a short close-range puff (1 sec) of 1% cVA did not evoke a significant response in *snmp1^1^* flies (Fig. 2D), a 20-second application of 1% cVA evoked a low level of spikes in *snmp1^1^* flies, which initiated after ∼7.5 seconds (Figure S2). This finding indicated that prolonged stimulation could partially compensate for the inadequacy of the low stimulation intensity in the *snmp1^1^* mutant flies.

On the basis of the findings here, we conclude that the so-called “spontaneous activity” exhibited by *snmp1^1^ Or67d* ORNs was not spontaneous neuronal activity. Instead, the action potentials were the result of extremely persistent cVA-induced activity, which remained long after removal of the cVA stimulus. Thus, SNMP1 was required not only for high cVA sensitivity, but also to achieve rapid on- and off-kinetics in response to cVA.

**Figure 4:**
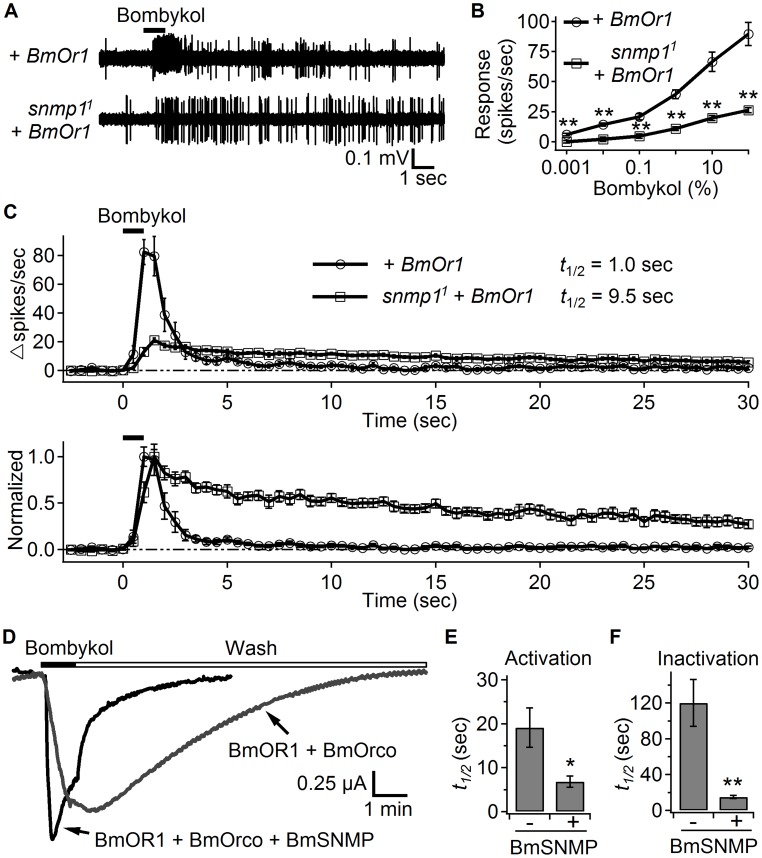
SNMP1 affects the response to bombykol in OR67d ORNs ectopically expressing BmOR1. We expressed *UAS-BmOr1* under control of the *snmp1-Gal4* in either a *snmp1^+^* or *snmp1^1^* background. We applied bombykol using the conventional odorant delivery approach, and performed SSRs from T1 sensilla from males and females. (A) Representative traces of bombykol-evoked responses from OR67d ORNs. We applied 100% bombykol for 1 sec as indicated by the horizontal black bar above the traces. (B) Dose-response curve for bombykol. n = 12–20 for each data point. (C) The upper graph shows the response dynamics of BmOR1-expressing OR67d neurons to bombykol. n = 14–15. The estimated *t*_1/2_ values are indicated. The lower graph shows the same traces from the upper panel normalized to their respective peak spiking rates. (D) Two-electrode voltage clamp recordings of Xenopus oocytes expressing BmOR1 and BmORCO with or without BmSNMP. 10 µM bombykol was applied as indicated by the black bar, and then removed (Wash) as indicated by the open bar. (E) Quantification of the times to reach 50% of the maximum activation (*t*_1/2_) during the 10 µM bombykol application. The results from cells with and without BmSNMP are indicated. n = 5–6. (F) Quantification of the times required for the decline to 50% of the maximum current (*t*_1/2_) after the washout of bombykol. n = 5–6. Means ±S.E.M. The asterisks indicate significant differences between groups (*p<0.05, **p<0.01). Unpaired Student's *t*-tests.

To address whether SNMP1 function was specific to either cVA or its receptor (OR67d), we tested whether SNMP1 affected the response to the silk moth (*Bombyx mori*) pheromone (E,Z)-10,12-hexadecadien-1-ol (bombykol), after we ectopically expressed the bombykol receptor, BmOR1 in OR67d ORNs (*UAS-BmOr1* and *OR67d^Gal4^*). As previously reported [14], [30], conventional application of bombykol to these transgenic flies evoked action potentials, which quickly terminated (Figure 4A). We then introduced BmOR1 in the *snmp1^1^* mutant background, and found that the ORNs still responded to bombykol applied by the conventional delivery method, but less robustly (Figure 4A and 4B). In addition, loss of SNMP1 slowed the activation and deactivation of the bombykol-evoked response (Figure 4C). Although the *snmp1^1^* mutation had a profound effect on deactivation, the phenotype was not as dramatic as with cVA. Consistent with this observation, pre-exposure of the transgenic flies to bombykol for 24 hours did not increase the basal firing rate (Figure S3). Nevertheless, the similar phenotypes after application of either cVA or bombykol suggested that SNMP1 functioned in the rapid activation and termination of pheromone-evoked neuronal activity.

Co-expression of BmOR1 and BmORCO is sufficient to form functional ion channels in Xenopus oocytes [31]. We took advantage of this *in vitro* reconstitution system to address whether SNMP1 directly affected the activation and inactivation of the pheromone receptor. We expressed the bombykol receptor complex, BmOR1 and BmORCO, either with or without the silk moth SNMP1 (BmSNMP) in Xenopus oocytes and performing two-electrode voltage clamp recordings. To quantify the kinetics of the bombykol response, we measured the half-time of the activation during bombykol application, and the half-time of the inactivation following the wash out of the pheromone. We found that upon introduction of the BmSNMP, the activation (*t*_1/2_) was nearly three-fold faster (Figure 4D and 4E; no BmSNMP, 19.1±4.5 seconds; +BmSNMP, 6.8±1.3 seconds). Moreover, the inactivation (*t*_1/2_) was accelerated eight-fold in the cells expressing BmOR1/BmORCO in combination with BmSNMP (Figure 4D and 4F; no BmSNMP, 120.2±26.3 seconds; +BmSNMP, 15.0±1.7 seconds). These results support a role for SNMP1 in directly accelerating receptor activation and inactivation in response to pheromone stimulation.

**Figure 5:**
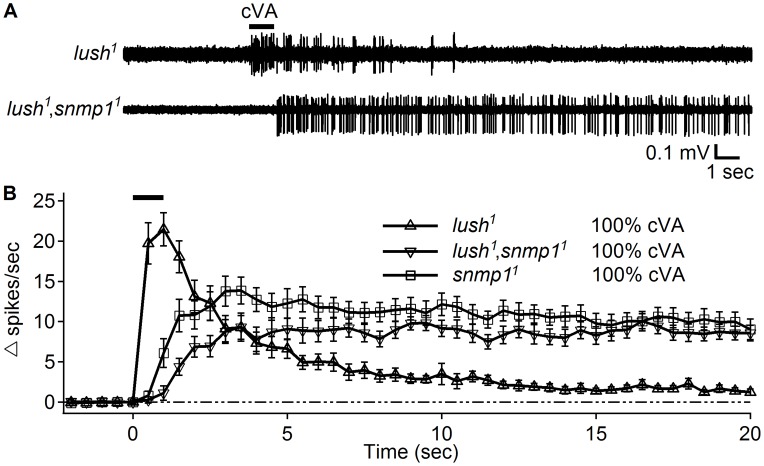
Close application of 100% cVA elicited responses in the *lush^1^*,*snmp1^1^* double mutants. (A) Representative traces of OR67d ORNs from the indicated genotypes in response to stimulation with 100% cVA applied at close range. The 1 sec application of cVA is indicated by the bar above the traces. (B) Response dynamics of OR67d ORNs from the indicated genotypes in response to 100% cVA applied using the close range approach (n = 14–18). *snmp1^1^* data were regenerated from Figure 2. Means ±S.E.M.

It has been reported that the OR67d ORNs from *lush^1^*,*snmp1^1^* double mutant flies also display high “spontaneous activity” [27], [28]. Therefore, we tested whether these action potentials were also due to very slow termination of the activity evoked by cVA. We first tested isolated *lush^1^* mutant females using the close-range application assay. Indeed, these flies responded to the 100% cVA stimulation (Figure 5A) as previously reported [26], and the response terminated within a few seconds after cessation of the stimulation. The *lush^1^*,*snmp1^1^* double mutant female flies raised in isolation did not show high spontaneous activity and also responded to the 100% cVA stimulation (Figure 5A). Similar to the *snmp1^1^* flies, the response from the *lush^1^*,*snmp1^1^* double mutant showed very slow termination kinetics that persisted after the stimulation. (Figure 5A and 5B).

## Discussion

Mutations that disrupt SNMP1 are reported to cause two impairments in OR67d ORNs in Drosophila [27], [28]. The first is insensitivity to cVA, and the second is increased spontaneous activity of OR67d ORNs in the absence of cVA stimulation. This latter phenotype motivated the proposal that the presence of SNMP1 somehow suppressed the spontaneous activity of OR67d [28].

Rapid termination is critical for an appropriate pheromone response, particularly for insects that use pheromones as tracking cues such as the silk moth, which relies on pheromone trails that are composed of intermittent odor pockets separated by clean air spaces [32]. Thus, to follow this trail, the pheromone-sensitive ORNs must quickly terminate their responses. It has been suggested that rapid inactivation of the pheromone response is due to degradation mediated by pheromone-degrading enzymes [23], [33], [34]. However, a mathematical model proposed that a soluble scavenger is required for the fast clearance of bombykol in the sensilla lymph, as enzymatic degradation may not be fast enough [35].

In the current work, we found that in contrast to previous studies, loss of SNMP1 neither eliminated cVA responsiveness nor caused high spontaneous activity. In support of these conclusions, *snmp1^1^* mutant females raised in isolation from males did not display elevated spontaneous activity. However, the *snmp1^1^* females exhibited high frequencies of action potentials if they were raised along with males, or if the isolated females were exposed to cVA prior to performing the recordings. The *snmp1^1^* mutation also did not eliminate cVA responsiveness, since the Or67d ORNs produced cVA-induced action potentials when we puffed the pheromone in close range to the mutant females. Thus, SNMP1 was not absolutely essential for OR67d ORN activation. This conclusion is supported by the finding that when OR67d is ectopically expressed in basiconic ORNs, which lack SNMP, the ORNs can be activated by cVA, if it is applied directly to the sensilla [29],

Of primary importance here, SNMP1 was required for rapid kinetic responses to cVA—both for rapid activation and termination of the responses. The pheromone-induced action potentials were dramatically delayed as they persisted for longer than 10 minutes, as opposed to ∼1 second for wild-type. Slow termination of cVA-induced responses also occurs upon introduction of SNMP1 antibodies to the recording pipet in wild-type flies [28]. We propose that the so-called spontaneous activity displayed by *snmp1^1^* null flies, was a consequence of extremely long-lived activity of OR67 ORNs following exposure to environmental cVA.

In addition to OR67d, ORCO and SNMP, a phospholipid flippase (dATP8B) and an OBP referred to as LUSH contribute to the sensitivity of ORNs to cVA. Loss of dATP8B affects the function of odorant receptors [36], [37], at least in part by decreasing the concentration of OR67d in the ORN dendrites [37]. However, the role of LUSH is controversial. While OBPs are typically thought to be carriers that transport hydrophobic odorants through the aqueous endolymph to the receptors [22], an *in vitro* study indicates that the cVA-LUSH complex is the activating ligand for OR67d [25]. This conclusion has recently been questioned, in part because OR67d neurons devoid of LUSH are activated by strong cVA stimulation *in vivo* [26]. Consistent with this latter report, we also found that cVA evoked responses in the *lush^1^* mutants and *lush^1^*,*snmp1^1^* double mutants if the pheromone was applied using the close-range application assay. Therefore, we favor the proposal that OR67d ORNs are activated directly by the pheromone.

SNMP1 function does not appear to be specific to cVA since the initiation and termination of the bombykol responses were also delayed in transgenic flies expressing the silk pheromone receptor, BmOR1. However, the delayed termination in the absence of SNMP1 was not as dramatic in response to bombykol as compared to cVA. The ORNs in T3 sensilla also express SNMP1 and respond to odors from fly bodies [27], [28], [29]. However, the T3 ORNs from wild-type or *snmp1^1^* males or females raised in groups or in isolation displayed similar basal activities. Thus, loss of SNMP1 does not always result in extremely prolonged activities in trichoid ORNs that are exposed to their ligands.

**Figure 6:**
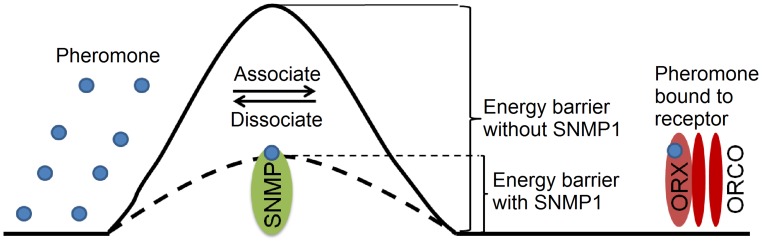
Proposed model for SNMP1 function in pheromone sensation. We suggest that SNMP1 promotes rapid activation and deactivation of the pheromone responses by lowering the energy barrier for the pheromone to associate and dissociate from the pheromone receptors (ORX).

A key question is whether SNMP1 regulates the pheromone response at the level of the receptors, or whether it modulates ORN activity downstream of receptor activation. To address whether SNMP1 activity modulated the response at the level of the receptors, we expressed the bombykol receptor complex in Xenopus oocytes, since this *in vitro* expression system was not likely to express other downstream signaling proteins that functioned in insect ORNs. We found that introduction of SNMP1 accelerated receptor activation by bombykol, and promoted rapid inactivation during wash out of the pheromone. A simple explanation for this result is that the pheromone binds to and dissociates from the receptor faster in the presence of SNMP1. We propose that SNMP1 facilitates the association and dissociation between ligands and receptors so that the receptor activation and inactivation are accelerated (Figure 6). On the surface, such a dual function might seem surprising, as association and dissociation are opposing processes. In this context it is noteworthy that an enzyme can increase both the forward and reverse reaction rates by lowering the activation energy of a reversible reaction.

The bombykol binding site in BmOR1 is proposed to consist of a large hydrophobic cavity buried between the transmembrane domains [38]. Thus, the interface between the hydrophilic sensillum lymph and the hydrophobic cavity inside the receptor might present a barrier preventing rapid on and off of the interaction between the pheromone and receptor. We suggest that SNMP1 helps overcome this barrier by facilitating the association and dissociation between the free pheromone in the sensillum lymph, and the hydrophobic pocket in the receptor (Figure 6). The barriers may vary among different receptors and thus the energy required for overcoming a barrier without SNMP1 might also be variable, potentially explaining why the severity of impairments resulting from loss of SNMP1 differ among odorant-receptors.

Finally, it is noteworthy that except for cVA, no other volatile pheromones are known in flies. In support of the existence of additional volatile pheromones, trichoid neurons other than T1 can be activated when fly cuticular extracts are released in their immediate proximity [29]. However, these neurons do not respond to the conventional odorant delivery assay. Tests for pheromone candidates other than cVA may have failed as a consequence of a lack of sensitivity provided by the conventional method for odorant delivery. Approaches that stimulate flies with high levels of pheromones may more closely replicate the situation in environments in which the animals are in close proximity, and may offer improved methods for identifying new volatile pheromones in Drosophila. These approaches include physically positioning odorants very close to the fly antenna [26], [29], or puffing the odorant close to the antenna as described here, which provides the additional advantage of more precise temporal control.

## Materials and Methods

### Drosophila strains

The mutant alleles and transgenic lines were: *Or67d^Gal4^*, *snmp1^1^*, *snmp1-Gal4*, *UAS-snmp1* [27], *lush^1^* [24] and *UAS-BmOr1* [30].

### Chemicals

cVA (99% purity) and bombykol (95% purity) were from Pherobank.

### Single sensillum recordings

#### Preparation of flies

To perform the recordings, we used flies that were 2–8 days old, unless indicated otherwise. The grouped flies were housed in populations of about 10 males and 10 females in typical food vials. To obtain isolated flies, we transferred individual pupae to small vials (10×50 mm). We used isolated female flies only to record cVA-evoked activity.

For experiments in which we pre-exposed flies to cVA or bombykol, and then measured ORN activity in the absence of acute cVA stimulation (Figure 1D), we prepared the flies as follows. We impregnated a small piece of filter paper with 5 µl of pheromones (either undiluted or diluted v/v in paraffin oil), and placed the filter paper in a vial with ordinary fly food. We then transferred 3–5 female flies (reared in isolation) into the vials for 24 hours. We performed the recordings within 20 min after removing the flies from the vials and the flies were sited under a charcoal-filtered and humidified air stream during the recording.

#### Odorant delivery

For the preparation of cVA or bombykol for stimulation, we applied 10 µl of the pheromones (either undiluted or diluted v/v in paraffin oil) to a filter paper, which we inserted into a glass Pasteur pipette. For performing the conventional odorant application assay, we injected the odorants by placing the odorant-containing pipet into a hole in the wall of a tube. We then diverted 50% of charcoal-filtered air (flowing at 36 ml/sec) through the odor pipette. The open end of the tube was positioned 15 mm away from the antenna and the airflow switch was under the control of the Syntech CS-55 stimulus controller. For the close-range application approach, we puffed cVA through a pipette, with the open tip placed 3 mm away from the antenna.

#### Recordings

We performed the single sensillum recordings essentially as described [30]. We used aluminosilicate glass electrodes inserted into the base of the sensilla. Signals were pre-amplified 10×, fed into a computer via a 16-bit analog-to-digital converter, and analyzed offline with AUTOSPIKE software (Syntech). To measure the activity in absence of cVA application, we kept the flies under a constant charcoal-filtered airflow to avoid potential exposure to environmental cVA, and counted the spikes over a 50 sec window. For cVA and bombykol stimulation, we recorded the signals starting 5 sec before initiating the odorant stimulation, and then continued to record for a total of 55 sec. We quantified the evoked responses (spikes/sec) by counting the spikes over a 0.5 sec window from the onset of the response, and subtracting the averaged basal firing rate per 0.5 sec before stimulation, based on quantification of spikes produced during the 5 sec window before stimulation. To determine the firing rates at different times following puff application of the pheromones, we subtracting the basal firing rates (activity prior to cVA stimulation) from the firing rates in 0.5 sec bins. The half-life *t*_1/2_ in deactivation was the first time point that the firing rate declined to ≤50% of the maximum firing rate before revoking the stimulation (based on the averaged traces of the kinetics). Following the prolonged (12 s) cVA stimulation, we determined the *t*_95_ for activation of the OR67d ORNs of *snmp1^1^* by identifying the time point in which the firing rate was ≥95% of the maximum frequency.

### Xenopus oocyte electrophysiology

#### Oocyte preparation

Pigmented *Xenopus laevis* females were housed at ∼18°C under 12 hr light/12 hr dark cycles at the Animal Resource Center (ARC) Bio II vivarium at the University of California, Santa Barbara (UCSB). All the procedures, including Xenopus surgery, ovary harvest, and post-surgical recovery, were performed according to a protocol approved by the institutional animal care and use committee at UCSB. Ovaries were surgically removed under anesthesia (0.3% w/v tricane, Sigma), cut into small pieces and then treated with 2 mg/ml collagenase A (Sigma-Aldrich C5138) in OR2 buffer (100 mM NaCl, 2 mM KCl, 1 mM 5 mM HEPES, pH 7.5) at room temperature until complete de-foliculation. The oocytes were recovered at 18°C for 12–24 hours in OR3 culture medium [50% Leibovitz's media L-15 (Sigma L1518), 13 mM HEPES, 90 g/ml gentamicin, 90 g/ml Fungizone (Amphotericin B), 90 g/ml penicillin/streptomycin, pH 7.5].

#### Oocyte injections

The *BmOr1* and *BmOrco* cDNAs, which were subcloned into the pXpBS2 vector, were kindly provided by Dr. Kazushige Touhara. We used RT-PCR to amplify the *Bmsnmp* cDNA from RNA isolated from silk moth antennae, and then subcloned the cDNA into the pXpBS2 vector. To prepare cRNAs for the oocyte injections, we linearized the plasmid templates, and performed *in vitro* transcription (mMESSAGE mMACHINE from Ambion). Purified cRNAs were mixed and injected into oocytes at the following final concentrations: *BmOr1* mRNA (0.2 µg/µl), *BmOrco* mRNA (0.2 µg/µl), *Bmsnmp* mRNA (0.4 µg/µl).

#### Oocyte recordings

We performed the two-electrode voltage clamp recordings on the third day post injection using a Xenoplace Workstation (ALA Scientific Instruments). Oocyte membrane potentials were clamped at −40 mV during the recordings. The electrodes were filled with 3 M KCl and displayed a resistance between 1–5 megaΩ. Channel currents were recorded using Turbo TEC-03X Two Electrode Clamp System, npi (Germany). The oocyte bath chamber was perfused with room temperature (∼22–25°C) ND96 recording buffer (96 mM NaCl, 2 mM KCl, 1 mM, 5 mM HEPES, 1.8 mM, pH 7.5). Bombykol was first dissolved in DMSO to generate a 1000× stock solution, and diluted with ND96 to a final concentration of 10 µM. A vehicle control ND96 solution was also prepared containing DMSO (1∶1000) and used during the recordings.

### Statistical analyses

The error bars represent SEMs. To assess statistical significance, we used the one-way ANOVA with Bonferroni-Holm *post hoc* analysis to compare multiple samples, and unpaired Student *t*-tests for comparing pairs of data.

## Supporting Information

Figure S1 Activity of T3 ORNs in singly and group housed wild-type and *snmp1^1^* males and females. (A) Representative trace showing firing activity from ORNs in a T3 sensillum without acute stimulation. A, B and C indicate three types of ORNs based on spike amplitudes. (B) Mean firing rates of the three types of ORNs in T3 sensilla. The genders and whether the flies were singly or group housed are indicated. Means ±S.E.M. n = 12–16.
(TIF)

Figure S2 Peak activities in responses to prolonged (20 sec) exposure of *snmp1^1^* mutants to cVA. (A) Representative trace showing the response of OR67d ORNs evoked by close-range application of 100% cVA for 20 sec (indicated by the horizontal bar above the trace). (B) Firing rates after close-range application of either 1% or 100% cVA for 20 sec as indicated by the black bar. Means ±S.E.M. n = 10–12.
(TIF)

Figure S3 Dependence on SNMP1 for basal spiking activity after pre-exposure to bombykol. We expressed *UAS-BmOR1* in OR67d neurons (*Or67d^Gal4^*) in an *snmp1^+^* or *snmp1^1^* background, and exposed the flies to 1 µl bombykol or the vehicle (paraffin oil) for 24 hrs immediately prior to the recordings. (A) Sample traces recorded from T1 sensilla. The recordings were performed without stimulation. (B) Average spiking frequencies from T1 sensilla without any stimulation during the recordings. Mean ±S.E.M. n = 16–18.
(TIF)
