# Supplementary figures and images for: Requirement for Drosophila SNMP1 for Rapid Activation and Termination of Pheromone-Induced Activity
Source: PLoS Genet. 2014 Sep 25;10(9):e1004600. doi: 10.1371/journal.pgen.1004600 (PMC4177743; doi:10.1371/journal.pgen.1004600)

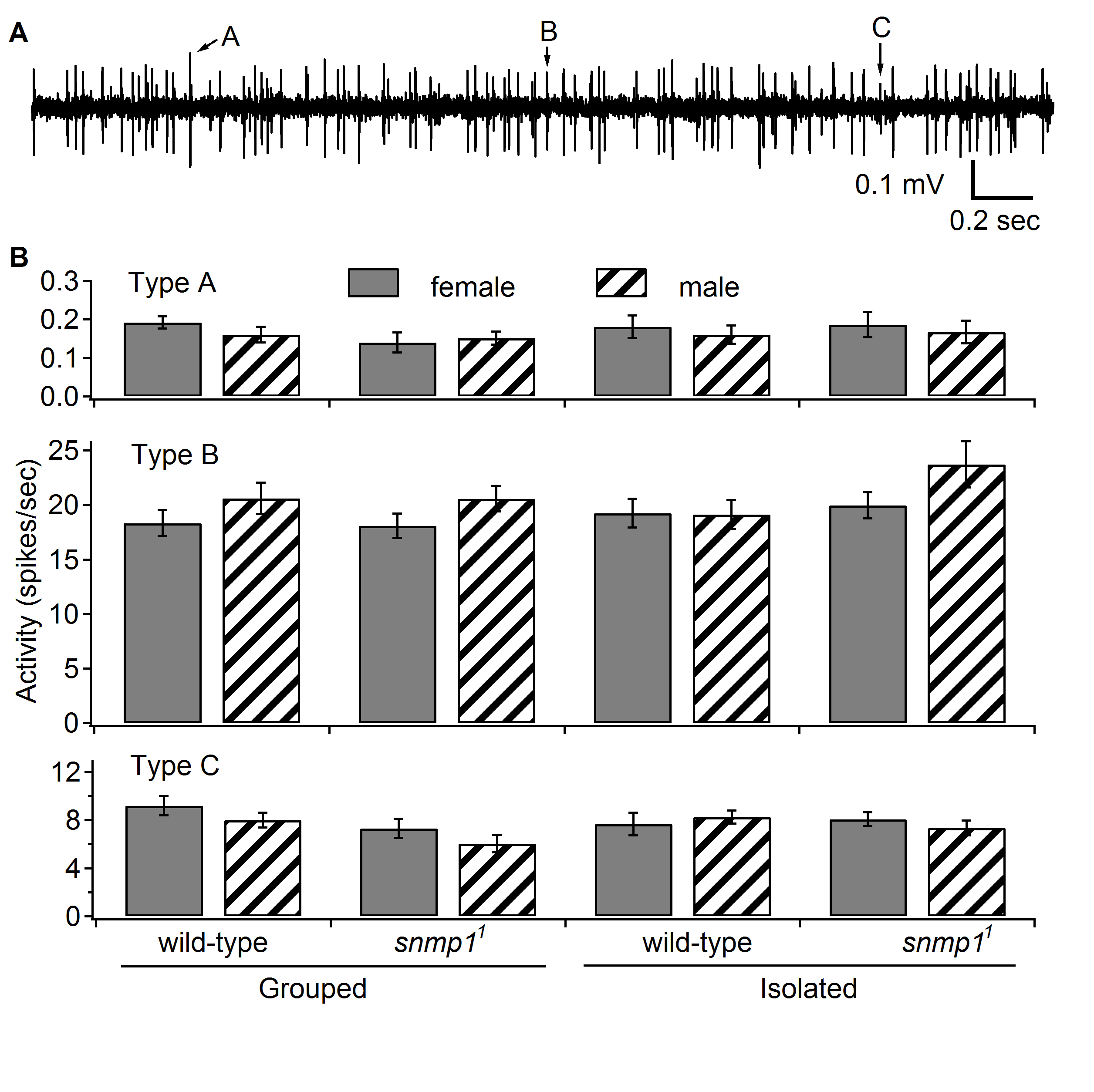

Supplement: Figure S1 — Activity of T3 ORNs in singly and group housed wild-type and snmp11 males and females. (A) Representative trace showing firing activity from ORNs in a T3 sensillum without acute stimulation. A, B and C indicate three types of ORNs based on spike amplitudes. (B) Mean firing rates of the three types of ORNs in T3 sensilla. The genders and whether the flies were singly or group housed are indicated. Means ±S.E.M. n = 12–16. (TIF) [file pgen.1004600.s001.tif]

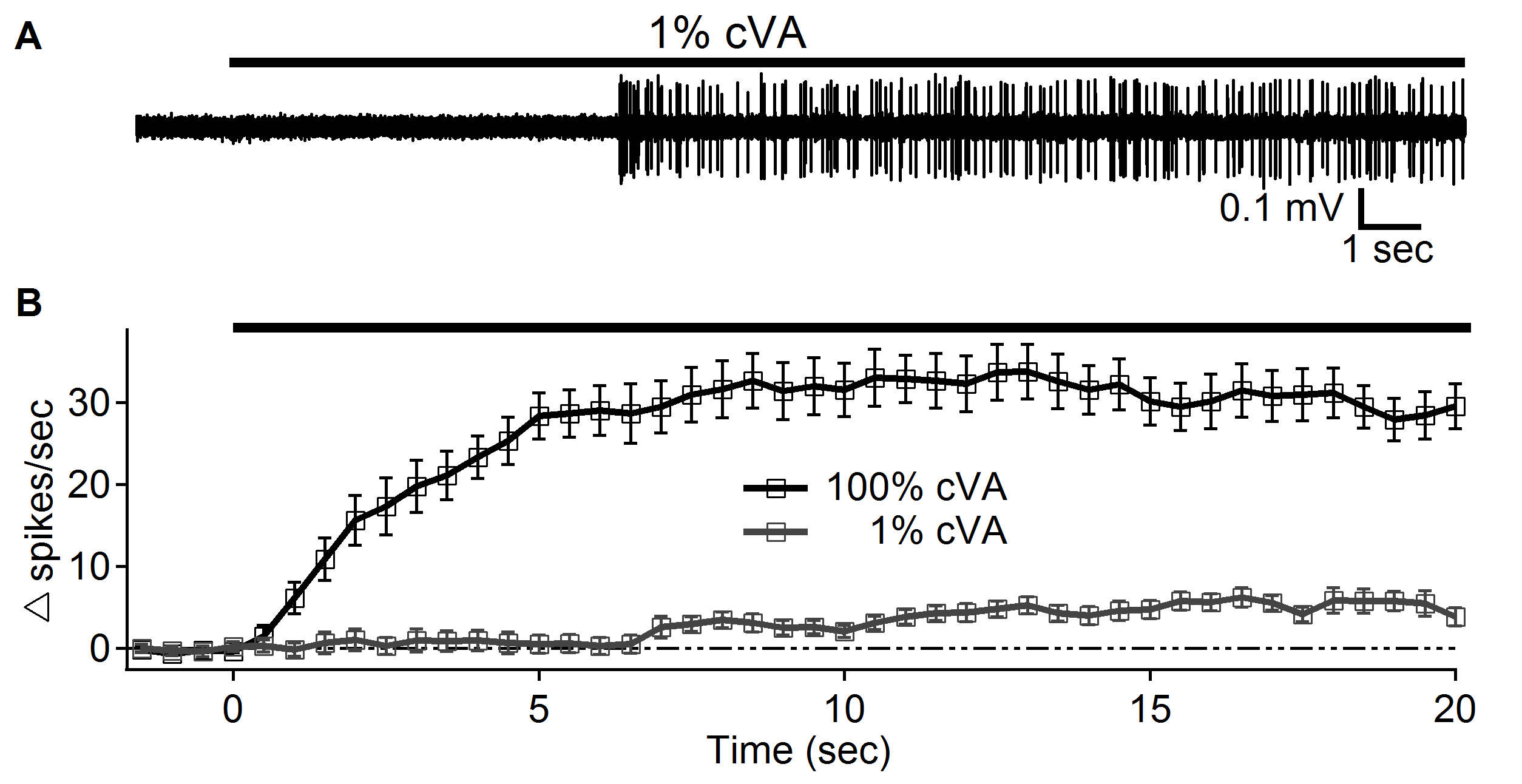

Supplement: Figure S2 — Peak activities in responses to prolonged (20 sec) exposure of snmp11 mutants to cVA. (A) Representative trace showing the response of OR67d ORNs evoked by close-range application of 100% cVA for 20 sec (indicated by the horizontal bar above the trace). (B) Firing rates after close-range application of either 1% or 100% cVA for 20 sec as indicated by the black bar. Means ±S.E.M. n = 10–12. (TIF) [file pgen.1004600.s002.tif]

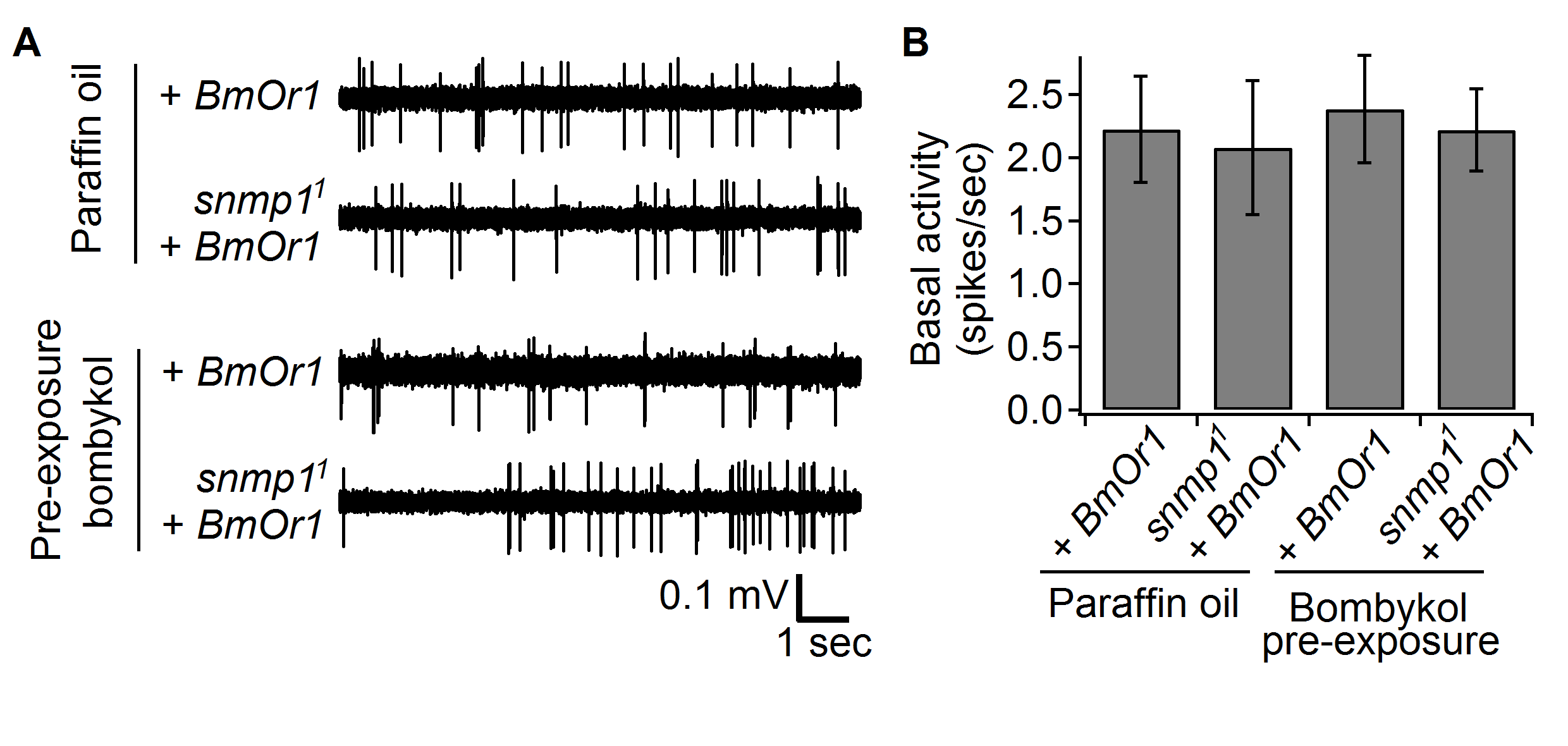

Supplement: Figure S3 — Dependence on SNMP1 for basal spiking activity after pre-exposure to bombykol. We expressed UAS-BmOR1 in OR67d neurons (Or67dGal4) in an snmp1+ or snmp11 background, and exposed the flies to 1 µl bombykol or the vehicle (paraffin oil) for 24 hrs immediately prior to the recordings. (A) Sample traces recorded from T1 sensilla. The recordings were performed without stimulation. (B) Average spiking frequencies from T1 sensilla without any stimulation during the recordings. Mean ±S.E.M. n = 16–18. (TIF) [file pgen.1004600.s003.tif]
